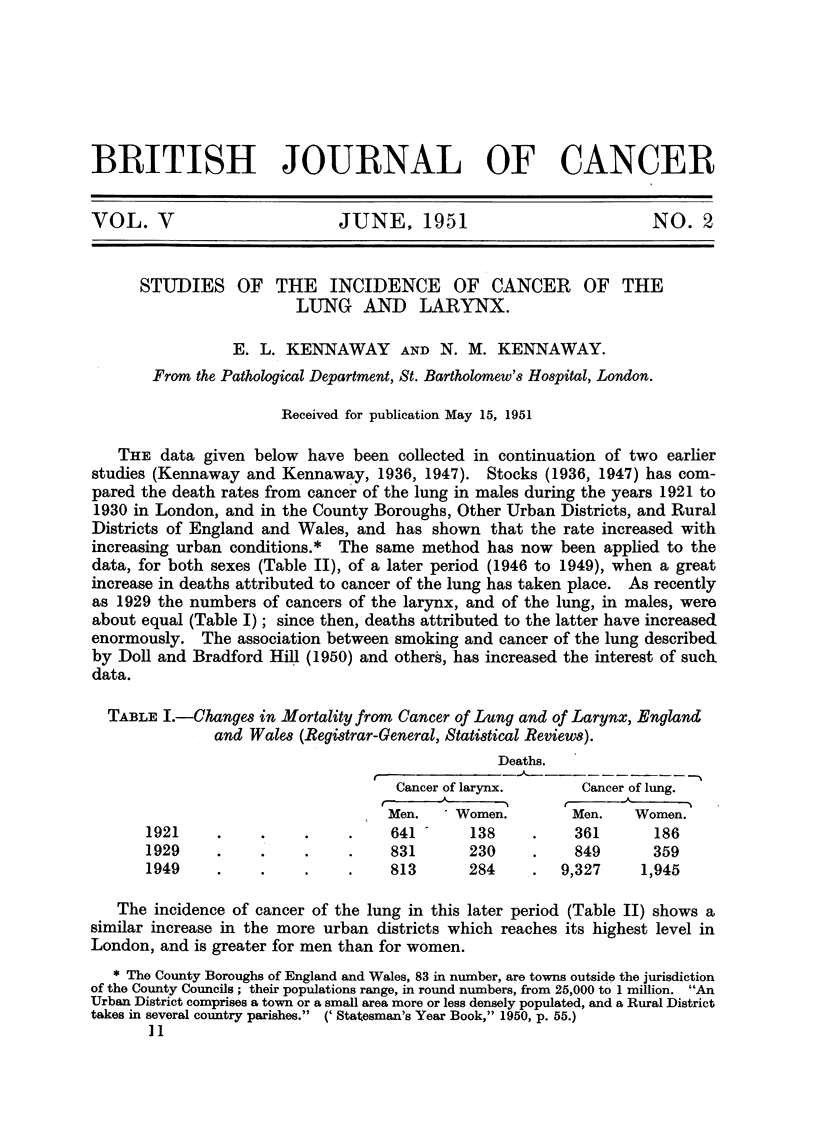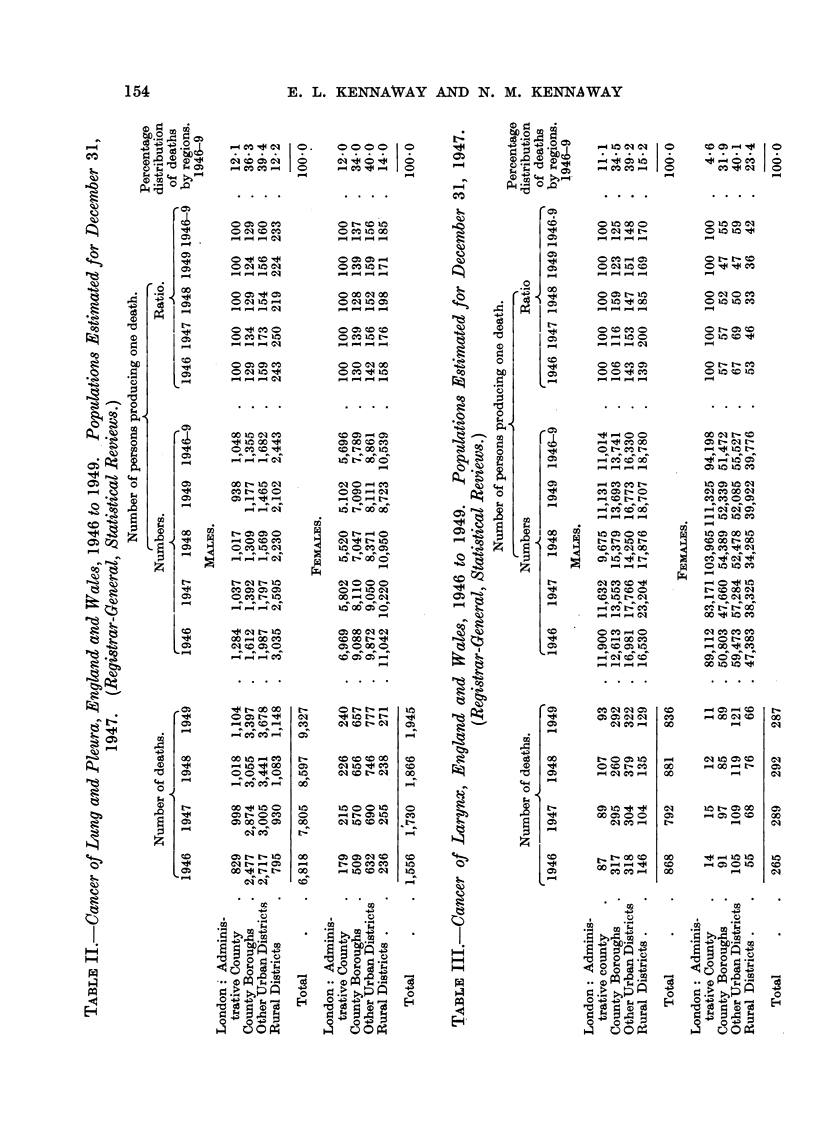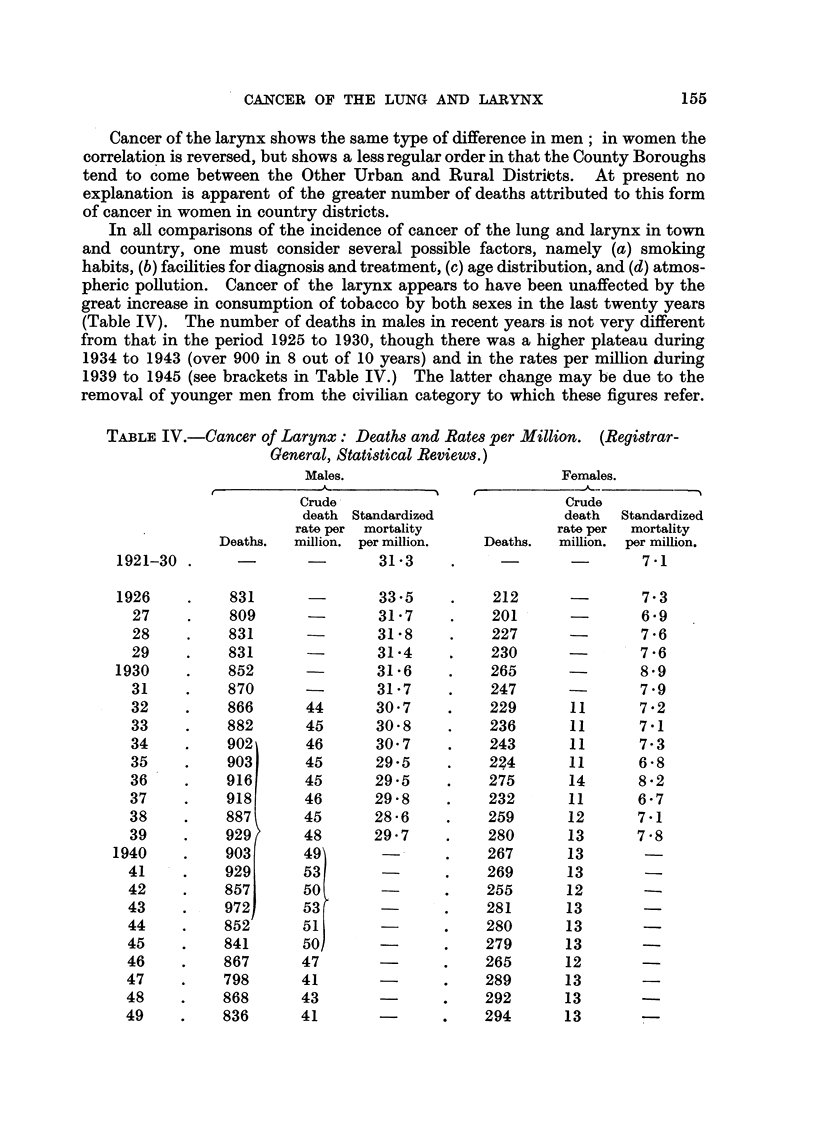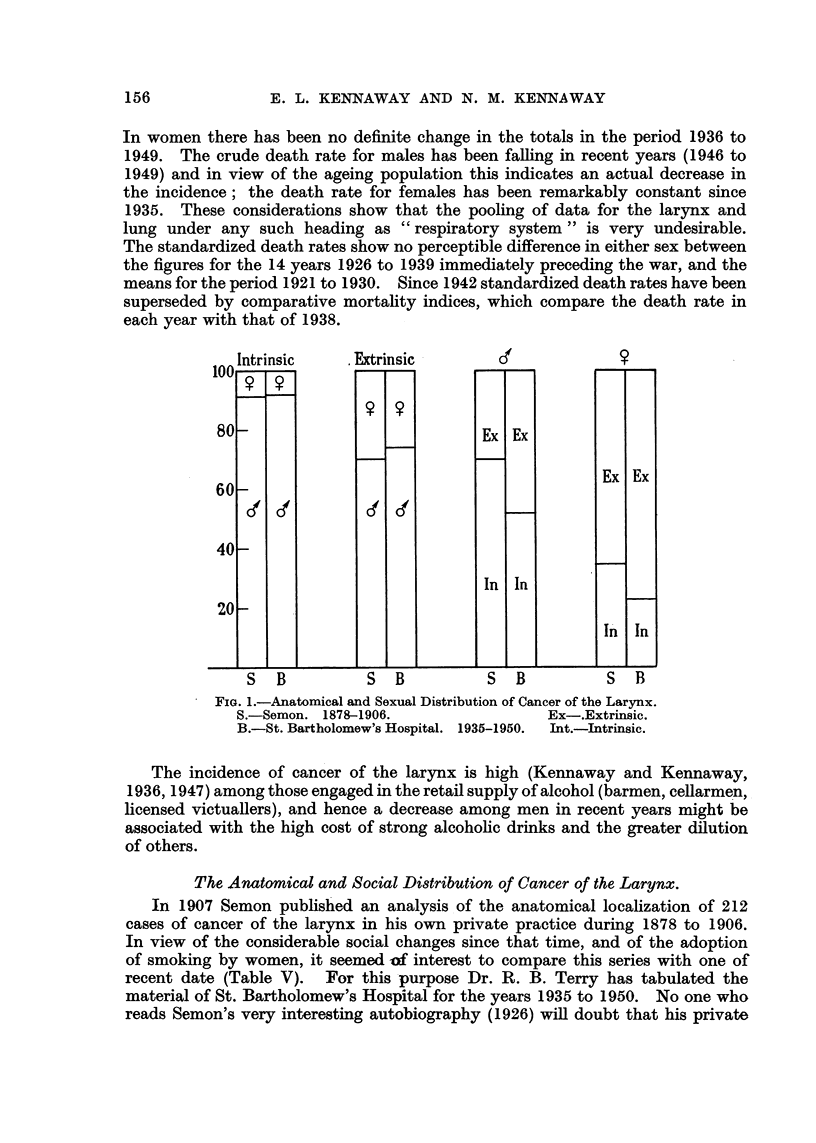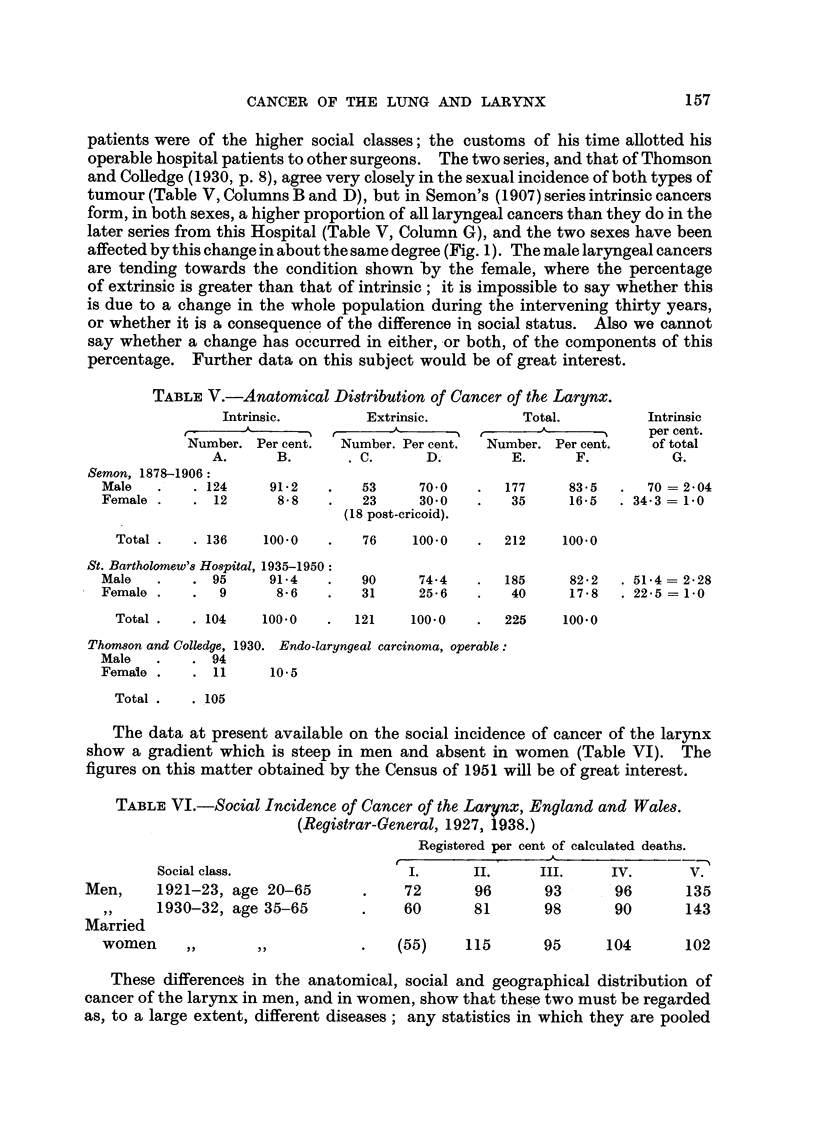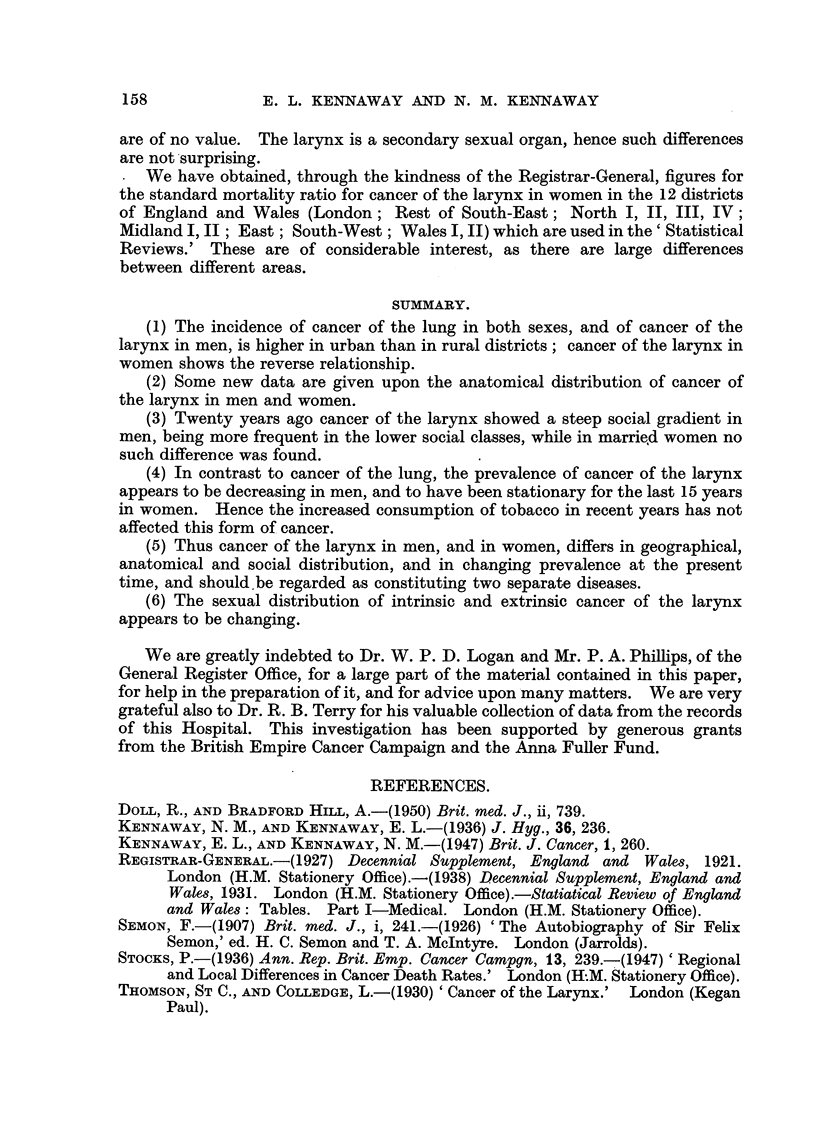# Studies of the Incidence of Cancer of the Lung and Larynx

**DOI:** 10.1038/bjc.1951.16

**Published:** 1951-06

**Authors:** E. L. Kennaway, N. M. Kennaway


// BRITISH JOURNAL OF CANCER

VOL. V JUNE, 1951 NO. 2

### STUDIES OF THE INCIDENCE OF CANCER OF THE LUNG AND LARYNX.

E. L. KENNAWAY AND N. M. KENNAWAY.

*From the Pathological Department, St. Bartholomew's Hospital, London.*

Received for publication May 15, 1951

THE data given below have been collected in continuation of two earlier studies (Kennaway and Kennaway, 1936, 1947). Stocks (1936, 1947) has compared the death rates from cancer of the lung in males during the years 1921 to 1930 in London, and in the County Boroughs, Other Urban Districts, and Rural Districts of England and Wales, and has shown that the rate increased with increasing urban conditions.* The same method has now been applied to the data, for both sexes (Table II), of a later period (1946 to 1949), when a great increase in deaths attributed to cancer of the lung has taken place. As recently as 1929 the numbers of cancers of the larynx, and of the lung, in males, were about equal (Table I); since then, deaths attributed to the latter have increased enormously. The association between smoking and cancer of the lung described by Doll and Bradford Hill (1950) and others, has increased the interest of such data.

TABLE I.—*Changes in Mortality from Cancer of Lung and of Larynx, England and Wales (Registrar-General, Statistical Reviews).*

| | Deaths. | | | |
|---|---|---|---|---|
| | Cancer of larynx. | | Cancer of lung. | |
| | Men. | Women. | Men. | Women. |
| 1921 | 641 | 138 | 361 | 186 |
| 1929 | 831 | 230 | 849 | 359 |
| 1949 | 813 | 284 | 9,327 | 1,945 |

The incidence of cancer of the lung in this later period (Table II) shows a similar increase in the more urban districts which reaches its highest level in London, and is greater for men than for women.

* The County Boroughs of England and Wales, 83 in number, are towns outside the jurisdiction of the County Councils; their populations range, in round numbers, from 25,000 to 1 million. "An Urban District comprises a town or a small area more or less densely populated, and a Rural District takes in several country parishes." (' Statesman's Year Book," 1950, p. 55.)

Cancer of the larynx shows the same type of difference in men ; in women the correlation is reversed, but shows a less regular order in that the County Boroughs tend to come between the Other Urban and Rural Districts. At present no explanation is apparent of the greater number of deaths attributed to this form of cancer in women in country districts.

In all comparisons of the incidence of cancer of the lung and larynx in town and country, one must consider several possible factors, namely (*a*) smoking habits, (*b*) facilities for diagnosis and treatment, (*c*) age distribution, and (*d*) atmospheric pollution. Cancer of the larynx appears to have been unaffected by the great increase in consumption of tobacco by both sexes in the last twenty years (Table IV). The number of deaths in males in recent years is not very different from that in the period 1925 to 1930, though there was a higher plateau during 1934 to 1943 (over 900 in 8 out of 10 years) and in the rates per million during 1939 to 1945 (see brackets in Table IV.) The latter change may be due to the removal of younger men from the civilian category to which these figures refer.

TABLE IV.—*Cancer of Larynx : Deaths and Rates per Million. (Registrar-General, Statistical Reviews.)*

| | Males. | | | Females. | | |
|---|---|---|---|---|---|---|
| | Deaths. | Crude death rate per million. | Standardized mortality per million. | Deaths. | Crude death rate per million. | Standardized mortality per million. |
| 1921–30 | — | — | 31·3 | — | — | 7·1 |
| 1926 | 831 | — | 33·5 | 212 | — | 7·3 |
| 27 | 809 | — | 31·7 | 201 | — | 6·9 |
| 28 | 831 | — | 31·8 | 227 | — | 7·6 |
| 29 | 831 | — | 31·4 | 230 | — | 7·6 |
| 1930 | 852 | — | 31·6 | 265 | — | 8·9 |
| 31 | 870 | — | 31·7 | 247 | — | 7·9 |
| 32 | 866 | 44 | 30·7 | 229 | 11 | 7·2 |
| 33 | 882 | 45 | 30·8 | 236 | 11 | 7·1 |
| 34 | 902 | 46 | 30·7 | 243 | 11 | 7·3 |
| 35 | 903 | 45 | 29·5 | 224 | 11 | 6·8 |
| 36 | 916 | 45 | 29·5 | 275 | 14 | 8·2 |
| 37 | 918 | 46 | 29·8 | 232 | 11 | 6·7 |
| 38 | 887 | 45 | 28·6 | 259 | 12 | 7·1 |
| 39 | 929 | 48 | 29·7 | 280 | 13 | 7·8 |
| 1940 | 903 | 49 | — | 267 | 13 | — |
| 41 | 929 | 53 | — | 269 | 13 | — |
| 42 | 857 | 50 | — | 255 | 12 | — |
| 43 | 972 | 53 | — | 281 | 13 | — |
| 44 | 852 | 51 | — | 280 | 13 | — |
| 45 | 841 | 50 | — | 279 | 13 | — |
| 46 | 867 | 47 | — | 265 | 12 | — |
| 47 | 798 | 41 | — | 289 | 13 | — |
| 48 | 868 | 43 | — | 292 | 13 | — |
| 49 | 836 | 41 | — | 294 | 13 | — |

In women there has been no definite change in the totals in the period 1936 to 1949. The crude death rate for males has been falling in recent years (1946 to 1949) and in view of the ageing population this indicates an actual decrease in the incidence; the death rate for females has been remarkably constant since 1935. These considerations show that the pooling of data for the larynx and lung under any such heading as "respiratory system" is very undesirable. The standardized death rates show no perceptible difference in either sex between the figures for the 14 years 1926 to 1939 immediately preceding the war, and the means for the period 1921 to 1930. Since 1942 standardized death rates have been superseded by comparative mortality indices, which compare the death rate in each year with that of 1938.

FIG. 1.—Anatomical and Sexual Distribution of Cancer of the Larynx.
S.—Semon. 1878–1906. Ex—.Extrinsic.
B.—St. Bartholomew's Hospital. 1935–1950. Int.—Intrinsic.

The incidence of cancer of the larynx is high (Kennaway and Kennaway, 1936, 1947) among those engaged in the retail supply of alcohol (barmen, cellarmen, licensed victuallers), and hence a decrease among men in recent years might be associated with the high cost of strong alcoholic drinks and the greater dilution of others.

*The Anatomical and Social Distribution of Cancer of the Larynx.*

In 1907 Semon published an analysis of the anatomical localization of 212 cases of cancer of the larynx in his own private practice during 1878 to 1906. In view of the considerable social changes since that time, and of the adoption of smoking by women, it seemed of interest to compare this series with one of recent date (Table V). For this purpose Dr. R. B. Terry has tabulated the material of St. Bartholomew's Hospital for the years 1935 to 1950. No one who reads Semon's very interesting autobiography (1926) will doubt that his private patients were of the higher social classes; the customs of his time allotted his operable hospital patients to other surgeons. The two series, and that of Thomson and Colledge (1930, p. 8), agree very closely in the sexual incidence of both types of tumour (Table V, Columns B and D), but in Semon's (1907) series intrinsic cancers form, in both sexes, a higher proportion of all laryngeal cancers than they do in the later series from this Hospital (Table V, Column G), and the two sexes have been affected by this change in about the same degree (Fig. 1). The male laryngeal cancers are tending towards the condition shown by the female, where the percentage of extrinsic is greater than that of intrinsic; it is impossible to say whether this is due to a change in the whole population during the intervening thirty years, or whether it is a consequence of the difference in social status. Also we cannot say whether a change has occurred in either, or both, of the components of this percentage. Further data on this subject would be of great interest.

TABLE V.—*Anatomical Distribution of Cancer of the Larynx.*

| | Intrinsic. | | Extrinsic. | | Total. | | Intrinsic per cent. of total |
|---|---|---|---|---|---|---|---|
| | Number. A. | Per cent. B. | Number. C. | Per cent. D. | Number. E. | Per cent. F. | G. |
| *Semon*, 1878–1906: | | | | | | | |
| Male | 124 | 91·2 | 53 | 70·0 | 177 | 83·5 | 70 = 2·04 |
| Female | 12 | 8·8 | 23 | 30·0 | 35 | 16·5 | 34·3 = 1·0 |
| | | | (18 post-cricoid). | | | | |
| Total | 136 | 100·0 | 76 | 100·0 | 212 | 100·0 | |
| *St. Bartholomew's Hospital*, 1935–1950: | | | | | | | |
| Male | 95 | 91·4 | 90 | 74·4 | 185 | 82·2 | 51·4 = 2·28 |
| Female | 9 | 8·6 | 31 | 25·6 | 40 | 17·8 | 22·5 = 1·0 |
| Total | 104 | 100·0 | 121 | 100·0 | 225 | 100·0 | |
| *Thomson and Colledge*, 1930. *Endo-laryngeal carcinoma, operable*: | | | | | | | |
| Male | 94 | | | | | | |
| Female | 11 | 10·5 | | | | | |
| Total | 105 | | | | | | |

The data at present available on the social incidence of cancer of the larynx show a gradient which is steep in men and absent in women (Table VI). The figures on this matter obtained by the Census of 1951 will be of great interest.

TABLE VI.—*Social Incidence of Cancer of the Larynx, England and Wales.* (*Registrar-General*, 1927, 1938.)

| | | Registered per cent of calculated deaths. | | | | |
|---|---|---|---|---|---|---|
| | Social class. | I. | II. | III. | IV. | V. |
| Men, | 1921–23, age 20–65 | 72 | 96 | 93 | 96 | 135 |
| ,, | 1930–32, age 35–65 | 60 | 81 | 98 | 90 | 143 |
| Married women | ,, ,, | (55) | 115 | 95 | 104 | 102 |

These differences in the anatomical, social and geographical distribution of cancer of the larynx in men, and in women, show that these two must be regarded as, to a large extent, different diseases; any statistics in which they are pooled are of no value. The larynx is a secondary sexual organ, hence such differences are not surprising.

We have obtained, through the kindness of the Registrar-General, figures for the standard mortality ratio for cancer of the larynx in women in the 12 districts of England and Wales (London; Rest of South-East; North I, II, III, IV; Midland I, II; East; South-West; Wales I, II) which are used in the 'Statistical Reviews.' These are of considerable interest, as there are large differences between different areas.

### SUMMARY.

(1) The incidence of cancer of the lung in both sexes, and of cancer of the larynx in men, is higher in urban than in rural districts; cancer of the larynx in women shows the reverse relationship.

(2) Some new data are given upon the anatomical distribution of cancer of the larynx in men and women.

(3) Twenty years ago cancer of the larynx showed a steep social gradient in men, being more frequent in the lower social classes, while in married women no such difference was found.

(4) In contrast to cancer of the lung, the prevalence of cancer of the larynx appears to be decreasing in men, and to have been stationary for the last 15 years in women. Hence the increased consumption of tobacco in recent years has not affected this form of cancer.

(5) Thus cancer of the larynx in men, and in women, differs in geographical, anatomical and social distribution, and in changing prevalence at the present time, and should be regarded as constituting two separate diseases.

(6) The sexual distribution of intrinsic and extrinsic cancer of the larynx appears to be changing.

We are greatly indebted to Dr. W. P. D. Logan and Mr. P. A. Phillips, of the General Register Office, for a large part of the material contained in this paper, for help in the preparation of it, and for advice upon many matters. We are very grateful also to Dr. R. B. Terry for his valuable collection of data from the records of this Hospital. This investigation has been supported by generous grants from the British Empire Cancer Campaign and the Anna Fuller Fund.

### REFERENCES.

DOLL, R., AND BRADFORD HILL, A.—(1950) *Brit. med. J.*, ii, 739.

KENNAWAY, N. M., AND KENNAWAY, E. L.—(1936) *J. Hyg.*, **36**, 236.

KENNAWAY, E. L., AND KENNAWAY, N. M.—(1947) *Brit. J. Cancer*, **1**, 260.

REGISTRAR-GENERAL.—(1927) *Decennial Supplement, England and Wales*, 1921. London (H.M. Stationery Office).—(1938) *Decennial Supplement, England and Wales*, 1931. London (H.M. Stationery Office).—*Statiatical Review of England and Wales*: Tables. Part I—Medical. London (H.M. Stationery Office).

SEMON, F.—(1907) *Brit. med. J.*, i, 241.—(1926) 'The Autobiography of Sir Felix Semon,' ed. H. C. Semon and T. A. McIntyre. London (Jarrolds).

STOCKS, P.—(1936) *Ann. Rep. Brit. Emp. Cancer Campgn*, **13**, 239.—(1947) 'Regional and Local Differences in Cancer Death Rates.' London (H.M. Stationery Office).

THOMSON, ST C., AND COLLEDGE, L.—(1930) 'Cancer of the Larynx.' London (Kegan Paul).